# Antihyperglycaemic treatment patterns, observed glycaemic control and determinants of treatment change among patients with type 2 diabetes in the United Kingdom primary care: a retrospective cohort study

**DOI:** 10.1186/1472-6823-14-73

**Published:** 2014-08-27

**Authors:** Andrew Maguire, Beth D Mitchell, Javier Cid Ruzafa

**Affiliations:** 1 Director Epidemiology and Database Analyses, OXON Epidemiology Ltd., The Euston Office, 1 Euston Square, 40 Melton Street, London NW1 2FD, UK; 2 Research Scientist, Global Patient Safety, Eli Lilly and Company, Indianapolis IN 46285, USA; 3 Research Scientist & Epidemiologist, Health Economics & Epidemiology, Evidera, Metro Building, 6th Floor, 1 Butterwick, London W6 8DL, UK

**Keywords:** Type 2 diabetes mellitus, Oral antidiabetics, Non-insulin antihyperglycaemic therapy, Treatment patterns, Population based, Cohort

## Abstract

**Background:**

The initial treatment strategy for patients with type 2 diabetes includes lifestyle change recommendations. When patients are not successful in controlling their blood glucose levels through healthier lifestyle pharmaceutical agents are recommended. The objective of this study is to identify determinants of initial treatment change following initiation of non-insulin antihyperglycaemic treatment (OAD) for UK patients with type 2 diabetes.

**Methods:**

A retrospective cohort study using primary care data from the Clinical Practice Research Datalink between January 2006 and February 2011. Each patient had an OAD prescription. The main treatment pattern outcomes were discontinuation, switching, augmentation and initiation of insulin. Glycaemic control was assessed using HbA1c.

**Results:**

63,060 patients initiated OAD therapy 2006–2010 and 3.4% were prescribed insulin during follow-up. 26% with at least four years of follow-up remained on the initial treatment. Metformin dominated (90%) in UK primary care. Around 75% had a record of HbA1c testing prior to initiating therapy. On initiating OAD, half the patients had HbA1c values >65 mmol/mol and one quarter >80 mmol/mol. The initial values of HbA1c were reduced after 12 months and remained stable. There were 15%-18% of patients whose values increased since initiating OAD. Increased baseline HbA1c is associated with increased chance of augmentation and decreased chance of discontinuation. HbA1c values at 1 year were associated with a three-fold increase in the chance of augmentation, 130% increase in the chance of switching and 14% increase in the chance of discontinuation with each 10 mmol/mol increase. Following initiation of OAD, HbA1c was reduced by an average of 16 mmol/mol during the first year.

**Conclusion:**

There are patients for whom glycaemic control worsens and a majority remained above the recommended level, suggesting an unmet need despite the availability of many OAD.

## Background

The incidence and prevalence of type 2 diabetes (T2D) in the United Kingdom (UK) has increased over recent years [1]. The aim of T2D treatment is to control blood glucose levels and to reduce the effects of chronic hyperglycaemia while avoiding serious hypoglycaemic events [2,3]. The effects of poor metabolic control results in increased cardiovascular risk, both at the macrovascular (i.e., coronary artery disease, peripheral artery disease, and carotid artery disease) and microvascular (i.e., retinopathy, nephropathy, and neuropathy) levels [4]. A commonly used measure of average blood glucose control is measuring glycated haemoglobin (HbA1c), which reflects plasma glucose control over the previous two to three months. Current UK recommendations aim to reach an HbA1c level of 6.5% (equivalent to 48 mmol/mol) or 7.5% (equivalent to 59 mmol/mol) for those at risk of severe hypoglycaemia, although these targets are not intended to be universally achieved [5]. There is evidence that direct medical costs of treating patients with T2D who have good glycaemic control are lower than those for patients who have fair or poor glycaemic control [6,7].

The initial treatment strategy for patients with T2D includes lifestyle change recommendations, involving exercise and reduced caloric intake aimed at obesity reduction and healthy weight maintenance. When patients are not successful in controlling their blood glucose levels through healthier lifestyle pharmaceutical agents are recommended [5,8]. In the UK the recommended and preferred prescribed oral antidiabetic (OAD) medications for the treatment of patients with T2D are metformin and sulfonylureas [1,5]. Alternative OAD and other non-insulin antihyperglycaemic treatment strategies have a variety of drug mechanisms and side-effect profiles that result in different costs and quality of life implications [9].

A review of publications from UK primary care has indicated that no study has been performed in recent years describing current treatment patterns and how they are associated to HbA1c. Since HbA1c has been consistently reported and recorded for several years now it seems timely to study antihyperglycaemic treatment patterns and their relationship with HbA1c. Our objective was to identify determinants of treatment change following initiation of non-insulin antihyperglycaemic treatment for UK patients with T2D.

## Methods

This was a retrospective cohort study using primary care data from the Clinical Practice Research Datalink (CPRD). CPRD is a large database which contains anonymized individual patient-level medical and demographic information on approximately 8% of the UK population from more than 630 general practices. The patient population captured in the database is broadly representative of the UK population and CPRD is a reliable resource for longitudinal epidemiological research [10].

The total study period was between January 1, 2006 and February 25, 2011 (end of data records). The follow-up period for each patient starts after the first non-insulin antihyperglycaemic drug is prescribed (index date) and ends at the earliest of the following: date of last data collection for the practice, date of transfer out (patient leaves the practice), date of probable pregnancy, date of death of the patient, or end of study period.

### Inclusion and exclusion criteria

We required that each patient had a prescription of antihyperglycaemic therapy (OAD or injectable) other than insulin between January 1, 2006 and December 31, 2010. Additionally, patients had to be permanently registered with a CPRD participating practice and have a valid date of birth and gender information (acceptable patients). Furthermore, all patients had to have at least 12 months of computerised data prior to the index date and the index date was later than the practice’s up-to-standard (UTS) date; this is the date from which CPRD considers the practices data to be sufficiently exhaustive to be used for research purposes.

We excluded patients pregnant at index date, with a specific diagnosis of type 1 diabetes mellitus at or prior to index date, a prescription of an OAD at any time prior to index date, a prescription of insulin prior to index date, who left the practice on index date or with a diagnosis of polycystic ovary syndrome (PCOS) if metformin is the index OAD (unless the patient was suffering both T2D and PCOS).

### Variables and outcomes

The main treatment pattern outcomes were discontinuation, switching, augmentation and initiation of insulin therapy. In order to define the treatment pattern outcomes at all times, it was necessary to censor each patient’s follow-up by 90 days since it is not possible to assess whether a patient has discontinued or not, or whether a switch has effectively occurred or not, if the last prescription occurs near the end of the patient’s records. Treatment patterns occurring subsequently to the first change in the OAD therapy are not in the scope of this project given the myriad of possible treatment patterns.

Duration of a prescription is key to defining treatment patterns. If the pack size or daily dose were missing for a prescription then the duration was imputed from typical durations observed for that drug or drug class. Failing this, and where appropriate, a value of 30 days was used.

#### Discontinuation

Discontinuation was firstly defined for the index OAD and then for all OADs; the latter allows for a patient to switch between OADs until all OAD therapy is discontinued. A patient was defined to have discontinued his/her index OAD if a gap occurs of at least 60 days between the expiry of the last prescription and the start of the next prescription of that particular OAD. For discontinuation of all OAD therapy, a gap of at least 60 days was again used irrespective of the type of OAD drug and whether switching in the OAD therapy had occurred during the initial period. Time to discontinuation (i.e., persistence) used the number of days from the index date until discontinuation date.

#### Switching & augmentation

A switch is defined when a new OAD replaces the previous one. For switching, diabetes therapy has to have been continuous, hence the new OAD (or insulin) has to start before the current OAD has been deemed to have discontinued. Hence, the difference between switching and augmentation is the degree of overlap between the two OAD therapies: if a new OAD is prescribed on or after the date of the last prescription of the index OAD (i.e., the overlap between the new and old drugs is less than the duration of one prescription) then we conclude that a switch has occurred, but only if the patients receives at least two prescriptions of the new drug. Augmentation occurs if the new OAD is introduced before the date of the last prescription. Again there must be at least two prescriptions of the new drug. Augmentation is defined as a prescription of a second OAD drug in addition to the index OAD without the discontinuation of the original drug.

#### Rates

The rates for discontinuation used as denominator time from index date until the earliest of either discontinuation or 90 days before end of follow-up whilst for switching and augmentation the follow-up time was terminated when the index OAD was discontinued. Initiation of insulin was assessed between index date and end of follow-up.

#### Other variables

We assessed variables of interest at baseline, sociodemographic information, comorbidities, OAD at index date (type and monotherapy or in combination) and laboratory test results including HbA1c (mmol/mol). Comorbidities included coronary heart disease (CHD), cerebrovascular disease, congestive heart failure (CHF), nephropathy, neuropathy, retinopathy, and hypertension, defined by the presence of the corresponding diagnostic or test Read codes [11]. In order to evaluate the trends in HbA1c, the HbA1c results were assigned to specific time periods. The baseline HbA1c was the most recent value recorded in the six months prior to index date. For each year since index date we assigned the HbA1c that was closest to the anniversary of the index date within the six months before or after this date.

### Statistical analysis

We described baseline characteristics of all study patients at the time of the first administration of an OAD. These characteristics included age, gender, HbA1c and other laboratory test results, relevant comorbidities and OADs, and other co-medication use. Numbers and percentages of patients were reported for categorical variables, and the mean and median with appropriate measures of spread were given for continuous variables.

Multinomial logistic regression models were applied to estimate the association between each covariate and the likelihood of each of the treatment outcomes as compared to no change in treatment during the timeframe of interest. The timeframes for which models were applied included the baseline period (first six months after index date) and yearly intervals since index. It was not possible to execute the models for time periods beyond one year since index date due to the decreasing numbers of patients over time.

Most covariates comprise of the baseline characteristics. However, HbA1c, age and time since diagnosis were updated to reflect time period being analysed. The most parsimonious models are reported.

Data programming, management and analyses were carried out using SAS (version 9.2).

The study was approved by the Independent Scientific Advisory Committee (ISAC) that provides advice to the Medicines and Healthcare Regulatory Agency on database research (ISAC protocol 10_186). The ISAC is a non-statutory expert advisory body to provide advice on research related requests to access data provided by CPRD (and data from the Yellow Card Scheme as well).

## Results

There were 63,060 patients who initiated non-insulin antihyperglycaemic therapy between 2006 and 2010. The median age of patients was 62 years old when they initiated their non-insulin antihyperglycaemic therapy and a little over half the patients were male. By definition this was the first time that these patients had been prescribed OAD. Around 75% of patients had a record of an HbA1c test prior to initiating OAD therapy, and 70% in the prior 6 months. There is a gradual increase over the study period in the proportion of this marker of glycaemic control. The prevalence of CHD was 16.8% and cerebrovascular disease was 6.5%. Retinopathy had a prevalence of 4.5%, compared to a prevalence of 0.7% for either nephropathy and neuropathy. Patient characterisation as tabulated for all patients is broken down according to initial OAD regimen in Table 1.

**Table 1:** Baseline patient characterization by main OAD at index date

	**OAD at index date**				Total*
	**Mono: metformin**	**Mono: gliclazide**	**Mono: glimepiride**	**Metformin & gliclazide**	
Number of patients (n)	55,522	5,132	417	927	63,060
Mean age at index date (years)	60.97	67.13	66.84	59.04	61.50
Gender (% male)	56.51	57.50	57.31	61.27	56.70
Median time from Dx diabetes to OAD initiation** (months)	11.9	10.5	12.17	0.77	11.63
Mean HbA1c in last 6 months (mmol/mol)	70.12	78.30	76.73	97.42	71.05
Median HbA1c in last 6 months (mmol/mol)	64.00	70.00	67.00	99.00	65.00
% of patients with HbA1c >59 mmol/mol	63.10	70.40	65.60	82.10	63.90
Median BMI	31.60	26.60	26.50	30.90	31.20
Coronary Heart Disease (%)	16.33	22.35	21.58	13.59	16.84
Cerebrovascular D’ (%)	6.04	10.76	11.75	6.47	6.48
Nephropathy (%)	0.56	1.73	0.72	0.11	0.66
Retinopathy (%)	4.47	4.35	7.43	1.83	4.45
Neuropathy (%)	0.64	0.80	0.96	0.22	0.65
CHF (%)	2.78	9.08	7.67	3.67	3.38
Hypertension*** (%)	54.74	51.79	50.84	43.04	54.23

*All patients included in the study hence summing to more than the four specific OAD groups.

**Duration is calculated from the time from first diagnostic code of diabetes if it occurs prior to initiation of OAD (some codes occurred later than OAD).

***Hypertension defined at any time prior to initiation of OAD therapy.

Approximately 90% of all patients received metformin as their initial treatment either as monotherapy or in combination with another OAD. The next most frequent first OAD was gliclazide which was prescribed to 9.6% and then glimepiride with just 0.8% (Table 2). Given that there were 19 different OADs, data on cohorts that initiate on any of the remaining 16 OADs (i.e., not metformin, gliclazide, or glimepiride) are sparse. Practically all patients (98.0%) started their OAD therapy as monotherapy, and 1.8% were administered two OADs. The remaining patients (0.2%) had been prescribed preformulated combinations whilst very few either had three OADs (13 patients) or a combination of a preformulated with another OAD (6 patients). Most of the combinations were metformin with gliclazide (85% of all combined OAD).

**Table 2:** Patient distribution according to initial OAD prescription and by year of first OAD administration

	**Year of first OAD administration**					**Total**
	**2006**	**2007**	**2008**	**2009**	**2010**	
Number of patients	11,564	12,277	12,419	13,251	13,549	63,060
Mono: metformin	84.4	87.1	88.1	89.3	90.7	88.1
Mono: gliclazide	10.3	9.0	8.5	7.6	5.9	8.1
Comb: metformin and gliclazide	1.2	1.2	1.7	1.5	1.7	1.5
Mono: glimepiride	1.1	0.8	0.6	0.5	0.4	0.7
Mono: other	1.3	0.7	0.4	0.3	0.6	0.6
Mono: glipizide	0.5	0.4	0.3	0.3	0.1	0.3
Comb: other	0.6	0.3	0.1	0.1	0.2	0.3
Mono: pioglitazone	0.4	0.2	0.2	0.2	0.2	0.2
Comb: metformin and other	0.2	0.2	0.1	0.2	0.2	0.2
Comb: metformin and glimepiride	0.1	0.1	0.1	0.1	0.1	0.1

A summary of the rates of each of the treatment changes for all patients over the first year of OAD treatment (with at least 15 months of follow-up) and for those in the four most common initial OAD groups, which represent 98% of all patients, is in Table 3. The initiation of insulin was very low during the first year. In this period 31% of patients discontinued their initial OAD therapy and 6% who switched to another therapy. The rate of discontinuation was lower for metformin than for the other two most common groups and initiation of insulin was higher in the three other groups.

**Table 3:** Treatment changes (%) over first year since initiation of therapy according to baseline OAD*

	**Overall**	**Monotherapy**			**Combination**
		**Metformin**	**Gliclazide**	**Glimepiride**	**Metformin & Gliclazide**
Discontinuation of index OAD	31.0 (30.5,31.4)	29.6 (29.2,30.1)	39.6 (37.9,41.3)	33.6 (28.2,39.2)	51.4 (47.2,55.7)
Discontinuation of all OAD	24.6 (24.2,25.0)	24.0 (23.6,24.5)	30.8 (29.2,32.4)	25.2 (20.3,30.5)	19.7 (16.5,23.3)
Switch of index OAD	6.1 (5.9,6.3)	5.8 (5.6,6.1)	8.0 (7.1,9.0)	8.7 (5.8,12.5)	2.9 (1.6,4.6)
Augmentation of index OAD	12.9 (12.6,13.3)	12.4 (12.1,12.7)	19.7 (18.3,21.1)	17.9 (13.6,22.6)	3.4 (2.1,5.3)
Started insulin	2.0 (1.8,2.1)	1.4 (1.3,1.5)	7.0 (6.1,7.9)	5.0 (2.8,8.2)	6.8 (4.9,9.2)

*Patients with at least 15 months of follow-up,% and 95% confidence intervals.

A total of 3.4% of all patients (n = 2,101) were administered insulin at some time during their follow-up. Among those who initiated insulin, 62% (n = 1,300) did so during the initial period of persistent OAD use, 32% were switched from OAD to insulin whilst 6% started insulin after having discontinued all OAD therapy (for at least 60 days). With regards to the initial insulin regimen, most patients (84%, n = 1,892) started on intermediate/long-acting insulin as compared to only 4.2% on short-acting insulin. The remainder (12%, n = 271) were administered both insulin types.

A summary of the rates of each of the treatment changes for all patients over the study follow-up is in Table 4. For patients with at least four years of follow-up the proportion of patients who remained on the initial treatment reduced to 26%. For these patients with at least four years of follow-up, 34% had discontinued their index OAD therapy.

**Table 4:** First change in treatment over the study follow-up since initiation of OAD*

	**<1 year**	**1–2 years**	**2–3 years**	**3–4 years**	**Over 4 years**
No change	74.1%	52.8%	41.0%	33.7%	25.6%
Discontinuation index OAD	16.2%	26.9%	31.3%	33.0%	34.0%
OAD augmentation	5.5%	13.4%	19.5%	24.6%	28.0%
Switch to other OAD	3.2%	5.5%	6.9%	7.3%	10.8%
Insulin starters:	1.0%	1.4%	1.3%	1.4%	1.6%
Switch to insulin	0.5%	0.8%	0.7%	0.6%	0.7%
OAD augmentation w/ insulin	0.4%	0.6%	0.6%	0.7%	0.8%
Augmented w/ insulin and OAD	0.0%	0.0%	0.0%	0.0%	0.0%
Switch to OAD and insulin	0.0%	0.0%	0.0%	0.0%	0.0%

*Patients with less than 90 days follow-up are excluded.

**Figure 1:**
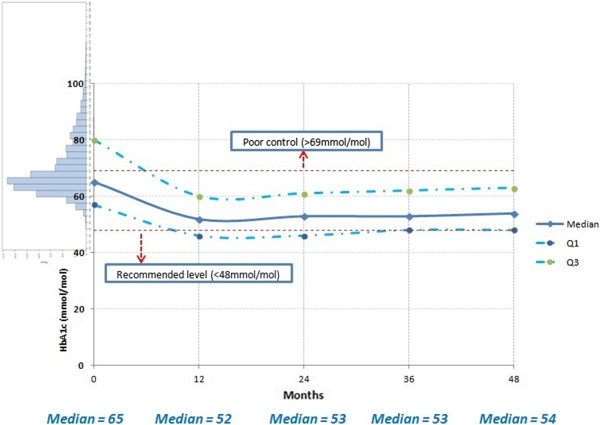
HbA1c (mmol/mol) values over time (in months) and 95% confidence intervals from baseline through four years of follow-up.

**Figure 2:**
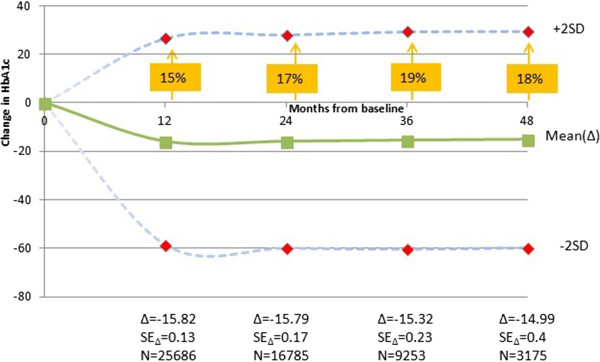
**HbA1c (mmol/mol) values (within-patient changes) over time (in months) and distribution of population values (95% of the population) from baseline through four years of follow-up (48 months).** Δ = change in HbA1c from baseline; SEΔ = standard error and SD = standard deviation of the change in HbA1c; N = number of patients.

The values of HbA1c are given in Table 5 and displayed in Figure 1 and Figure 2. For the entire patient group, the initial high values of HbA1c of around 71 mmol/mol were reduced by 12 months to 55 mmol/mol and remained at similar (lower) levels over the following years. Whilst Figure 1 depicts absolute HbA1c values for all patients, Figure 2 provides the within patient change in HbA1c values since baseline. Whilst most patients have benefited in a reduction of HbA1c, Figure 2 provides evidence that there was between 15% and 18% of patients for whom HbA1c increased since initiating OAD.

**Table 5:** HbA1c values over time since index date

**HbA1c (mmol/mol)**	**N**	**Mean**	**SD**	**SE**	**Median**	**Q1**	**Q3**
At baseline (during 6 months prior to index date)	43,863	71.05	20.86	0.10	65	57	80
At 12 months (+/- 6 months)	34,737	55.05	14.27	0.08	52	46	60
At 24 months (+/- 6 months)	22,650	55.72	14.51	0.10	53	46	61
At 36 months (+/- 6 months)	12,631	56.58	14.92	0.13	53	48	62
At 48 months (+/- 6 months)	4,383	57.53	15.29	0.23	54	48	63

SD = standard deviation; SE = standard error.

The association from multinomial logistic regression between each covariate and the likelihood of each of the treatment outcomes as compared to no change in treatment during the timeframe, for the period between 6 months and 18 months, is presented in Table 6. This period is potentially of greatest interest as the initial period reflects simply the baseline status of the patients whilst the second period around one year reflects how the patient has evolved since starting OAD, principally with respect to glycaemic control, and how the current status impacts of the chance of treatment change.

**Table 6:** Factors associated with treatment change* occurring between 6–18 months following OAD initiation

**Treatment change**	**Covariates**	**Odds ratio change/No change**	**95% CI**		**Statistical significance**
			**Lower**	**Upper**	
Augmentation	10 mmol/mol inc. in HbA1c	2.798	2.691	2.910	p < 0.05
	10-year increase in age	0.862	0.827	0.900	p < 0.05
	1-year increase in time since Dx	0.946	0.926	0.967	p < 0.05
	Male vs. female	1.021	0.923	1.129	NS
	Baseline OAD (vs. metformin)				
	Gliclazide	1.632	1.339	1.988	p < 0.05
	Glimepiride	2.554	1.458	4.474	p < 0.05
	Glipizide	1.251	0.496	3.160	NS
	Pioglitazone	1.293	0.414	4.037	NS
	Other monotherapy	2.111	1.017	4.384	p < 0.05
	Metformin w/ gliclazide	0.470	0.215	1.027	NS
	Metformin w/ glimepiride	NC			
	Metformin w/ other OAD	1.540	0.192	12.371	NS
	Other combination	0.310	0.040	2.375	NS
	Comorbidity				
	CHF	1.585	0.999	2.515	NS
Switch (any)	10 mmol/mol inc. in HbA1c	2.373	2.249	2.503	p < 0.05
	10-year increase in age	1.005	0.939	1.075	NS
	1-year increase in time since Dx	0.982	0.952	1.012	NS
	Male vs. female	0.809	0.690	0.948	p < 0.05
	Baseline OAD (vs. metformin)				
	Gliclazide	1.575	1.165	2.130	p < 0.05
	Glimepiride	1.842	0.721	4.705	NS
	Glipizide	2.490	0.879	7.052	NS
	Pioglitazone	7.734	3.131	19.101	p < 0.05
	Other monotherapy	5.882	2.855	12.119	p < 0.05
	Metformin w/ gliclazide	1.860	0.875	3.956	NS
	Metformin w/ glimepiride				
	Metformin w/ other OAD	16.929	5.208	55.023	p < 0.05
	Other combination	11.679	5.337	25.560	p < 0.05
	Comorbidity				
	CHF	1.222	0.562	2.659	NS
Discontinuation	10 mmol/mol inc. in HbA1c	1.139	1.092	1.187	p < 0.05
	10-year increase in age	0.886	0.854	0.919	p < 0.05
	1-year increase in time since Dx	1.006	0.991	1.022	NS
	Male vs. female	0.978	0.897	1.065	NS
	Baseline OAD (vs. metformin)				
	Gliclazide	1.762	1.500	2.071	p < 0.05
	Glimepiride	0.883	0.439	1.774	NS
	Glipizide	1.112	0.430	2.875	NS
	Pioglitazone	1.738	0.754	4.007	NS
	Other monotherapy	1.430	0.721	2.836	NS
	Metformin w/ gliclazide	1.795	1.207	2.668	p < 0.05
	Metformin w/ glimepiride	3.959	0.926	16.921	NS
	Metformin w/ other OAD	2.491	0.791	7.844	NS
	Other combination	0.576	0.176	1.884	NS
	Comorbidity				
	CHF	1.709	1.196	2.443	p < 0.05

*Treatment change refers to first change: period of reference is 6 to 18 months following initiation of OAD.

All variables were statistically significant in multinomial logistic regression. Other covariates not significant and hence not included are: CHD, retinopathy, nephropathy, neuropathy, and BMI.

CI = confidence interval.

### Augmentation

Increased baseline HbA1c is associated with increased chance of augmentation, and there is about a three-fold increase in the chance of augmentation with each 10 mmol/mol (equivalent to 0.9%) increase in HbA1c around one year after initiation of OAD therapy. Both increasing age and increasing time since diagnosis of diabetes are associated with decreased chance of augmentation, and there is no clear association of gender on the chance of augmentation. Metformin had a lower chance of augmentation than the other OADs provided in monotherapy as the initial pharmacological option. Patients on metformin were less likely to experience augmentation than those on gliclazide or glimepiride irrespective of medical history or HbA1c test results (since they are adjusted for in the model). For some of the groups administered OAD as combined therapy it would also appear that the rate of augmentation was lower than those who initiated on metformin. Patients with a history of CHF seemed to be more likely to have their treatment augmented in the period around one year following initiation of OAD therapy.

### Switching

The chance of switching increased by a factor of 2.4 with each 10 mmol/mol increase in HbA1c as measured around one year after initiating OAD therapy. There was no clear association of age and time since diagnosis of diabetes on the chance of switching. However, the chance of switching was observed to be lower in men. The chance of switching was lowest in the metformin group as compared with all the other initial OAD regimens, including those in combination though not all comparisons reached statistical significance.

### Discontinuation

Discontinuation implies that no switch to another OAD or initiation of insulin occurred shortly after the discontinuation. Hence, this variable implies that no further medication for diabetes was used for at least 60 days following exhaustion of the last OAD prescription. Increased baseline HbA1c was related to a decreased chance of discontinuation (for each increase of 10 mmol/mol of HbA1c the chance of discontinuation decreases by approximately 20%, regression coefficient = 0.805, data not shown). However, this association is inverted for HbA1c measurements in the period around 1 year after initiation of OAD. In this later period there is statistically significant increase in the chance of discontinuation associated with increased HbA1c whereby for each increase of 10 mmol/mol of HbA1c the chance of discontinuation increases by approximately 14%.

## Discussion

We report the results of a study that describes the determinants of the second-line treatment for over 60,000 UK patients with T2D, who initiated non-insulin antihyperglycaemic therapy between January 2006 and December 2010, for whom a median of two years of follow-up per patient was obtained, reaching a maximum of over 5 years. In the UK, T2D is principally managed in primary care. Therefore, an evaluation of treatment patterns in a primary care database will be generalizable to the overall population of patients with diabetes in the UK. Furthermore, this is a timely study as there is now enough information on HbA1c to analyse the changes in glycaemic control over time.

Whilst we examined how HbA1c can impact on subsequent changes in treatment regimen, this study also provided information on the levels at baseline when OAD was initially prescribed. Given that HbA1c is a key part of the guidelines related to when to initiate OAD and the choice of OAD, it was surprising that around 30% of patients did not have a recorded HbA1c test performed in the six months prior to initiating OAD. The gradual trend of increased rates of HbA1c recording over time may indicate that missing HbA1c may in part be due to lack of computerisation. However, even at the end of the study period 23% of patients were still lacking a record of HbA1c prior to commencing OAD. We observed that half of the patients initiating OAD had HbA1c values in excess of 65 mmol/mol and a quarter were above 80 mmol/mol. Such values are well above the National Institute for Health and Care Excellence (NICE) recommended level for OAD indication (48 mmol/mol). This would imply that in primary care either patients are being identified at high HbA1c levels (first OAD reading) or, in general, general practitioners are more likely to delay initiation of OAD until levels are often above the NICE recommendation for OAD. Over half of the patients that initiated OAD were clinically obese. This observation requires careful interpretation as this study only includes patients starting OAD and hence does not includes those T2D patients who have not required OAD and for whom lifestyle advice has been effective enough to avoid medication to control their glycaemia. Hence, the high prevalence of obesity in this population is a probable reflection that for these patients lifestyle modifications have not had the desired effect.

Metformin clearly dominated the choice of OAD in UK primary care between 2006 and 2010. This is in line with NICE recommendations. The key difference between the patients whose initial OAD was not metformin as compared to those that initiated on metformin, was that they were older and had worse levels of blood glucose. Prior to initiating gliclazide patients had a mean HbA1c of 78.3 mmol/mol, as compared to 70.1 mmol/mol for patients who were subsequently administered metformin. Body mass index (BMI) was lower for those patients who did not initiate with metformin. The prevalence of comorbidities (CHD, stroke, CHF) were all substantially higher for those patients who initiated on drugs other than metformin, suggesting certain channelling towards different OAD therapies. Indeed, it is likely the comorbidities may have dictated the choice of drugs giving rise to the older age of the patients prescribed sulfonylureas (rather than age determining directly the choice of OAD) and it is also possible that the choice of a therapy is based on the predominant pathophysiology in this group where older and less obese patients are likely to have more beta cell dysfunction and relatively less insulin resistance. The above differences were coherent with treatment guidelines. For instance, metformin is recommended especially for patients with high BMI whilst it is contraindicated for heart failure, (recent) myocardial infarction and for renal impairment. Whilst relatively low, the prevalence of nephropathy was three times less for metformin than for gliclazide. Initiating on combination therapy was rare (2%). Only 3.4% of all patients eventually initiated insulin during the follow-up period. Studies from other settings have reported a different preferred first-line OAD. In New England, US, during the 90 days after initial diabetes documentation 25% of patients received one oral antidiabetic agent (76.6% of them sulfonylurea and 21.9% metformin) [12]. Similarly, the ADVANCE trial reported that at baseline 43% of patients were on oral monotherapy glucose lowering agent, 59% on sulfonylureas, and 38% on metformin [13]. A study conducted in PHARMO for the period 1999–2004 also reported sulfonylureas (53.2%) and metformin (40.2%) as the most frequently used initial OAD therapies [14]. Metformin use as first OAD therapy seems to be increasing in recent years in other countries as well [15].

The within-patient reduction of 15.8 mmol/mol in the first year provides a quantification of the impact on the overall population of patients with diabetes. However, whilst this is a clear reduction a majority of patients remain above the recommended level (48 mmol/mol or 6.5%) and do so over the subsequent years. Furthermore, whilst demonstrating and quantifying the effectiveness of OAD administration, the observed overall reduction at the patient level actually masks the fact that there are around a fifth of all patients for whom their HbA1c levels actually increase. Hence, both issues suggest an unmet need in spite of the many non-insulin antihyperglycaemic drugs currently available. Indeed, further research is warranted to identify potential determinants of patients for whom a reduction in HbA1c is not achieved. Despite the clear improvement of HbA1c following OAD initiation, the overall levels of glycaemic control were poor with three quarters of the patients above 48 mmol/mol after three years. This is similar to other studies, in the New England region of the US, at 180 days after initial diabetes documentation, only 25% of the patients had optimal glycaemic control (defined as HbA1c <53 mmol/mol) [12].

The characteristics of patients who initiated on one of the main OAD drugs (i.e., metformin or a sulfonylurea) varied considerably. Such characteristics may, therefore, have an impact on subsequent glycaemic control and hence on the subsequent treatment regimen changes. All treatment changes were clearly associated with higher HbA1c levels, including discontinuation. This latter observation does not seem to be coherent as discontinuation would be associated with adequate glycaemic control; however, there will be patients restarting a different OAD shortly after discontinuing. When comparing the one-year persistence of initial OAD treatment with other published studies there was similarity with some studies [14,16], whilst there were clear differences with others [15]. The range of persistence rates can be explained by the settings where the studies were conducted, by the outcome definitions applied, and by the length of follow-up considered for the reported treatment changes. Another contributing factor to treatment changes could be the frequency of adverse events experienced by patients in different OAD therapies [17].

For this manuscript we reported the results of the multinomial logistic regression and in the interest of space we decided not to report the survival analyses that we also conducted.

Multinomial logistic regression provides intuitive results when patients can have one of several interrelated outcomes (i.e. switch, augmentation or discontinuation vs. persistence) whilst survival analyses are limited to one single event and censors all other outcomes (i.e. discontinued vs. did not discontinue). We were also able to account for the change in HbA1c, as a predictor for treatment pattern change using the HbA1c associated with the timeframe in which the change occurred, in a simpler way than if survival analyses were used only considering one outcome at a time.

A limitation of using logistic regression is that variable follow-up is not accounted for. Nevertheless, the patients who were included in these analyses all had to have had at least 15 months of follow-up and the outcome is specifically defined in the time window comprising 6 months to 18 months since OAD initiation. Hence in order to be considered the patients have to comply with similar criteria regarding their follow-up.

A potential limitation is that data analysis taking into consideration clustering by general practice would have provided insight into potential bias resulting from variable data quality and confidence intervals that could have a different width than reported. We find reassuring that others have reported little evidence of such potential bias after matching on practice [18]. Likewise, the use of matching on general practice could result in wider confidence intervals but it could also reduce variability overall [19].

Another limitation of the study is the lack of a comparator group; we cannot say what would have happened to the levels of blood glucose if the patients had not been treated although it is unlikely that without medication their HbA1c levels would have reduced given that life style counselling had already be tried. Another limitation we encountered, during multinomial logistic regression analyses, is that there were inferences hampered by the low numbers.

## Conclusion

This study confirmed the high rate of initiation on metformin and apparent channelling when other agents were used, as well as that treatment regimen changes were related to HbA1c levels following OAD initiation. We also observed that when patients started their first OAD their levels tended to be substantially higher than those at which OAD drug use is indicated. Furthermore, this study provides a quantification of the reduction of HbA1c at the diabetes patient population level, although the levels remained above those recommended and there are many patients for whom their HbA1c levels increased.

## Abbreviations

BMI: Body mass index; CHF: Congestive heart failure; CPRD: Clinical practice research datalink; NICE: National Institute for Health and Care Excellence; OAD: Oral antidiabetic; PCOS: Polycystic ovary syndrome; T2D: Type 2 diabetes; UK: United Kingdom; US: United States; UTS: Up-to-standard.

## Competing interests

This study was commissioned by Eli Lilly and Company. Ms Mitchell is an Eli Lilly employee and reports personal fees from Eli Lilly and Company, during the conduct of the study and personal fees from Eli Lilly and Company, outside the submitted work.

Drs Maguire and Cid Ruzafa report a grant from Eli Lilly and Company for the conduct of the study; Dr Cid Ruzafa reports personal fees from Bayer as former employee, and Drs Maguire and Cid Ruzafa report grants from Eli Lilly and Company, for other projects outside the submitted work. They are researchers independent from Eli Lilly and Company, who conduct research projects commissioned by a variety of sponsors through their affiliation to research and consulting companies that partner with life sciences organizations worldwide and are driven by world-class science.

## Authors’ contributions

Dr AM contributed to the study design, acquisition, analysis and interpretation of data, drafted the manuscript submitted for publication and approved the final version to be published. Ms BDM contributed to the study design, interpretation of data, revised the manuscript submitted for publication and approved the final version to be published. Dr JCR contributed to the interpretation of data, drafted the manuscript submitted for publication and approved the final version to be published. All authors read and approved the final manuscript.

## Authors’ information

AM (MSc, FSS) Director, Epidemiology at OXON Epidemiology.

BDM (RN, BSN, MPH student) Research Scientist, Global Patient Safety at Eli Lilly and Company.

JCR (MD, DrPH, MBA, MSc) Research Scientist and Epidemiologist, Health Economics and Epidemiology at Evidera.

## Pre-publication history

The pre-publication history for this paper can be accessed here:

http://www.biomedcentral.com/1472-6823/14/73/prepub
